# BRD2 inhibition blocks SARS-CoV-2 infection in vitro by reducing transcription of the host cell receptor ACE2

**DOI:** 10.1101/2021.01.19.427194

**Published:** 2021-01-19

**Authors:** Ruilin Tian, Avi J. Samelson, Veronica V. Rezelj, Merissa Chen, Gokul N. Ramadoss, Xiaoyan Guo, Alice Mac Kain, Quang Dinh Tran, Shion A. Lim, Irene Lui, James Nunez, Sarah J. Rockwood, Na Liu, Jared Carlson-Stevermer, Jennifer Oki, Travis Maures, Kevin Holden, Jonathan S. Weissman, James A. Wells, Bruce Conklin, Marco Vignuzzi, Martin Kampmann

**Affiliations:** 1 Institute for Neurodegenerative Diseases, Department of Biochemistry and Biophysics, University of California, San Francisco, San Francisco, CA 94158, USA; 2 Chan-Zuckerberg Biohub, San Francisco, CA 94158, USA; 3 Present address: School of Medicine, Southern University of Science and Technology, Shenzhen, China 518055; 4 Institut Pasteur, Viral Populations and Pathogenesis Unit, CNRS UMR 3569, 75015 Paris, France; 5 Gladstone Institutes, San Francisco, 94158, CA, USA; 6 Biomedical Sciences PhD Program, University of California, San Francisco, CA, USA; 7 École Doctorale BioSPC, Université de Paris, Sorbonne Paris Cité, 75006 Paris, France; 8 Department of Pharmaceutical Chemistry, University of California San Francisco, San Francisco, California, 94158, USA; 9 Present address: Department of Antibody Engineering, Genentech Inc., South San Francisco, CA, 94080, USA.; 10 Department of Cellular and Molecular Pharmacology, University of California, San Francisco, CA 94158, USA.; 11 Howard Hughes Medical Institute, University of California, San Francisco, CA 94158, USA; 12 Synthego Corporation, Redwood City, CA 94063, USA; 13 Whitehead Institute for Biomedical Research, Cambridge, 02142, USA; 14 Department of Biology, Massachusetts Institute of Technology, Cambridge, 02142, USA; 15 Innovative Genomics Institute, Berkeley, 94720, CA, USA; 16 Department of Ophthalmology, University of California, San Francisco, San Francisco, CA. 94158, USA; 17 Department of Medicine, University of California, San Francisco, San Francisco, CA, 94158, USA; 18 Department of Biochemistry and Biophysics, University of California, San Francisco, San Francisco, CA, 94158, USA

## Abstract

SARS-CoV-2 infection of human cells is initiated by the binding of the viral Spike protein to its cell-surface receptor ACE2. We conducted an unbiased CRISPRi screen to uncover druggable pathways controlling Spike protein binding to human cells. We found that the protein BRD2 is an essential node in the cellular response to SARS-CoV-2 infection. BRD2 is required for ACE2 transcription in human lung epithelial cells and cardiomyocytes, and BRD2 inhibitors currently evaluated in clinical trials potently block endogenous ACE2 expression and SARS-CoV-2 infection of human cells. BRD2 also controls transcription of several other genes induced upon SARS-CoV-2 infection, including the interferon response, which in turn regulates ACE2 levels. It is possible that the previously reported interaction between the viral E protein and BRD2 evolved to manipulate the transcriptional host response during SARS-CoV-2 infection. Together, our results pinpoint BRD2 as a potent and essential regulator of the host response to SARS-CoV-2 infection and highlight the potential of BRD2 as a novel therapeutic target for COVID-19.

## Introduction

The ongoing COVID-19 pandemic is a public health emergency. As of January 2020, SARS-CoV-2, the novel coronavirus causing this disease, has infected 95 million people worldwide, causing at least two million deaths, and these numbers are rapidly increasing. There is still a major need to elucidate the molecular mechanisms that underlie how SARS-CoV-2 interacts with host cells. A more detailed understanding will enable the development of therapeutics to treat and prevent COVID-19, complementing ongoing vaccination efforts.

SARS-CoV-2 entry into human cells is initiated by the interaction of the viral Spike protein with its receptor on the cell surface, Angiotensin-converting enzyme 2 (ACE2). To uncover new therapeutic targets targeting this step of SARS-CoV-2 infection, we conducted a focused CRISPRi-based screen for modifiers of Spike binding to human cells. We expected that ACE2 and factors regulating ACE2 expression would be major hit genes in this screen. A second motivation for identifying regulators of ACE2 was the fact that ACE2 affects inflammatory responses and is itself regulated in the context of inflammation^1–3^. Inflammatory signaling, in particular the type I interferon response, is known to be misregulated in the most severely affected COVID-19 patients^4–7^. Therefore, regulators of ACE2 expression would likely be relevant for COVID-19 in human patients, as suggested by clinical data^8^.

Previous CRISPR screens were performed in cell-based models of SARS-CoV-2 infection that overexpressed an ACE2 transgene^9,10^, represented cell types not primarily targeted by SARS-CoV-2^11^, or were non-human cells^12^. While these studies elucidated major features of SARS-CoV-2 biology, we reasoned that the cell lines used would not have enabled the discovery of regulators of ACE2 expression in relevant human cell types.

Here, we selected a lung epithelial cancer cell line, Calu-3, which endogenously expresses ACE2, to perform a targeted CRISPRi screen to find novel regulators of Spike protein binding. We found that the strongest hit genes are potent regulators of ACE2 levels. Knockdown of these genes reduced or increased ACE2 levels transcriptionally, and prevented or enhanced, respectively, SARS-CoV-2 infection in cell culture. We identified the transcriptional regulator BRD2 as a major node for host-SARS-CoV-2 interaction. BRD2 was necessary for ACE2 expression in Calu-3 cells, iPSC-derived cardiomyocytes and primary human lung epithelial cells. Inhibition of BRD2 with small molecules, some of which are in phase I clinical trials, completely abrogated SARS-CoV-2 infection in cell culture. We found that BRD2 is a direct regulator of ACE2 and of a number of interferon-induced genes that are upregulated during COVID infection. We propose BRD2 as a key regulator and therapeutic target for COVID-19.

## Results

### CRISPRi Screen for determinants of Spike-RBD binding to human cells

To identify cellular mechanisms controlling the binding of SARS-CoV-2 to human cells, we first aimed to identify a cell line that would robustly bind the viral spike protein (S). Our goal was to use a cell line that would not require exogenous overexpression of an ACE2 transgene, because this would prevent us from identifying mechanisms regulating endogenous ACE2 expression.

**Extended Data Figure 1:**
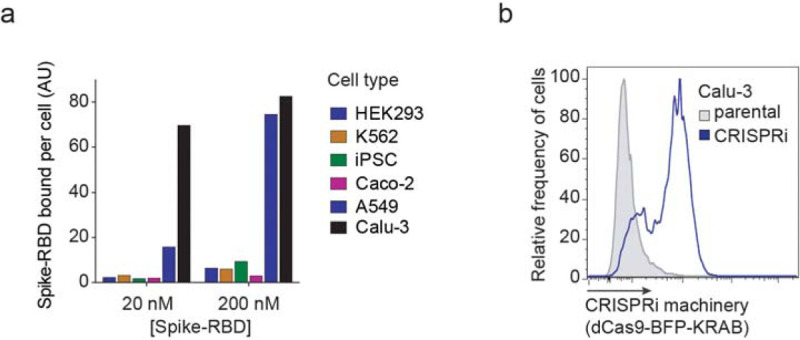
Calu-3 cells bind Spike-RBD specifically and were engineered to express CRISPRi machinery **a**, Spike-RBD binding in different cell types at 20 nM and 200 nM Spike-RBD was quantified by flow cytometry. **b**, Expression of CRISPRi machinery (dCas9-BFP-KRAB) in the CRISPRi Calu-3 line indicated by the expression of BFP by flow cytometry.

**Figure 1:**
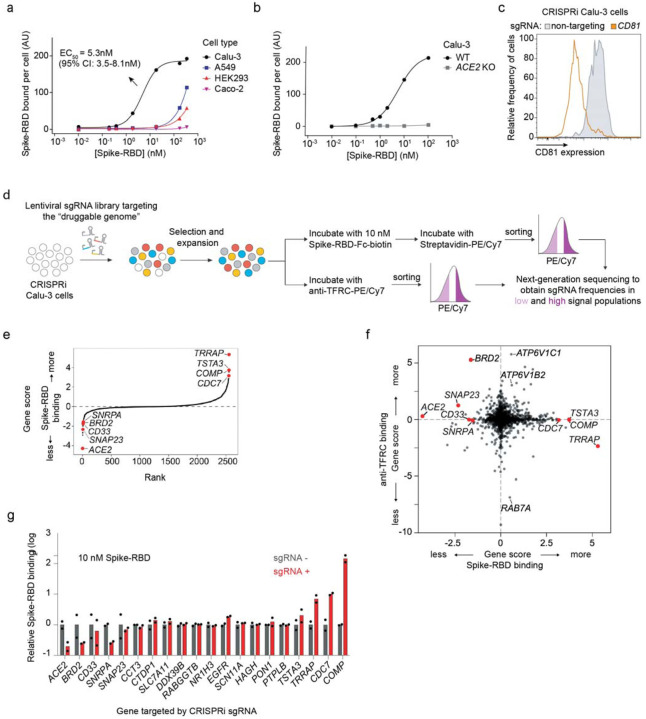
CRISPRi screen reveals cellular factors controlling Spike protein binding **a**, Human cell lines were incubated with different concentrations of recombinant SARS-CoV-2 Spike protein receptor-binding domain (Spike-RBD) and the amount of Spike-RBD bound per cell was quantified by flow cytometry. Only Calu3 cells (black) display saturable binding, for which an EC_50_ value was fit. Error of EC_50_ is the 95% confidence interval. **b**, Spike-RBD binding is eliminated in Calu-3 cells with ACE2 knockout (grey). **c**, Validation of CRISPRi activity of a Calu-3 CRISPRi line. Calu-3 cells stably expressing CRISPRi machinery were transduced with an sgRNA targeting CD81 (orange) or a non-targeting sgRNA (grey), and CD81 levels were determined by flow cytometry. **d**, CRISPRi screen strategy. CRISPRi Calu-3 cells transduced with an sgRNA library targeting the “druggable genome” are stained either with Spike-RBD or an anti-TFRC antibody. Cells are then FACS-sorted into bins based on Spike-RBD or anti-TFRC binding, and frequencies of cells expressing each sgRNA are determined for each bin by targeted next-generation sequencing. **e**, Rank-order plot of Spike-RBD hit genes. Genes selected for follow-up experiments are highlighted as red dots. **f**, Scatter plot of gene scores for the Spike-RBD screen (x-axis) and the anti-TFRC screen (y-axis). Genes selected for follow-up experiments are highlighted as red dots. **g**, Validation of hit genes using individually cloned sgRNAs. For each gene, the fold change of Spike-RBD binding at 10 nM Spike-RBD relative to cells not expressing sgRNAs is shown. Columns represent the average of two biological replicates (individual values shown as dots).

We measured binding of a previously described recombinant protein construct encompassing the SARS-CoV-2 Spike protein receptor-binding domain with a C-terminal human IgG Fc-domain fusion^13^, referred to hereafter as Spike-RBD, to several commonly used human cell lines (Fig. 1a and Extended Data Fig. 1a). Only Calu-3 cells displayed a binding curve consistent with specific binding of Spike-RBD, with an EC_50_ of 5.3 nM (95% CI: 3.5 – 8.1 nM). This value agrees with the dissociation constant of Spike-RBD-ACE2 binding determined *in vitro*, 4.7 nM^14^, within measurement error. Spike-RBD binding is dependent on ACE2 expression, as binding is completely abrogated in Calu-3 ACE2 knockout cells (Fig. 1b). Calu-3 cells are a particularly attractive model for studying SARS-CoV-2, as they are derived from lung epithelia, which is selectively infected by SARS-CoV-2 in patients^15^, are known to support infection of SARS-CoV and SARS-CoV-2^16^ and have recently been reported to closely recapitulate gene expression changes that occur in patients^17^.

We next generated a polyclonal Calu-3 line constitutively expressing machinery to enable CRISPR interference (CRISPRi)-based genetic screens^18,19^ and validated its CRISPRi activity (Fig. 1c and Extended Data Fig. 1b). Using this line, we then performed a focused CRISPRi screen for factors controlling Spike-RBD binding (Fig. 1d). In order to maximize our chances of identifying potential novel therapeutic targets for COVID-19, we screened a sgRNA library targeting the “druggable genome”, comprising ~2,300 genes, with ~16,000 total sgRNAs including non-targeting control sgRNAs^20^. In parallel, we screened the same library using a fluorophore-conjugated antibody against the transferrin receptor (TFRC, also known as CD71), to control for factors that generally affect protein trafficking or protein binding to the cell surface (Fig. 1d–f).

As expected, *ACE2* was the strongest hit knockdown of which decreased binding of Spike-RBD, while having no effect on TFRC levels (Fig. 1e,f). Conversely, *RAB7A*, which was recently reported to be essential for the trafficking of TFRC to the cell surface^21^, was the strongest hit that decreased TFRC levels, with no effect on Spike-RBD binding (Fig. 1f). Generally, hits were not correlated between the two screens (Fig. 1f), demonstrating the specificity of each screen. While the screens did not result in a large number of strong hits (Extended Data Table 1), we decided to validate the top 15 genes knockdown of which decreased Spike-RBD binding and the top five genes knockdown of which increased Spike-RBD binding. We cloned individual sgRNAs targeting each of these genes and evaluated their effect on Spike-RBD binding (Fig. 1g). Based on these experiments, we selected five hits that robustly recapitulated their phenotypes from the primary screen for further characterization: two genes knockdown of which decreased Spike-RBD binding (*ACE2* and *BRD2*), and three genes knockdown of which increased Spike-RBD-binding (*CDC7*, *COMP* and *TRRAP*).

### Hit genes modulate ACE2 levels and affect infection with SARS-CoV-2

**Figure 2:**
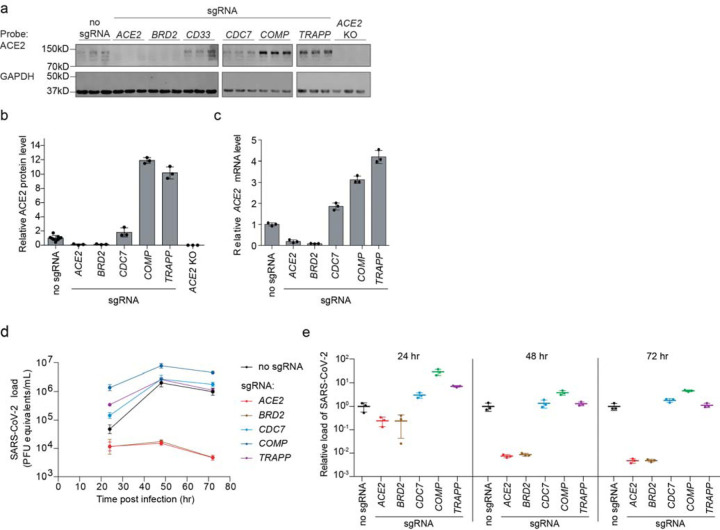
Hit genes modulate ACE2 levels and SARS-CoV-2 infection **a**, Western blotting for ACE2 and GAPDH in Calu-3 CRISPRi cells expressing no sgRNA or sgRNAs targeting different hit genes, or ACE2 knockout Calu-3 cells. Three lanes represent biological triplicates for each cell line. **b**, Quantification of ACE2 protein levels relative to GAPDH based on the data in (a). Average and standard deviation for three biological replicates are shown. **c**, Relative amounts of ACE2 transcript levels measured by qPCR in Calu-3 CRISPRi cells expressing sgRNAs targeting different hit genes, compared to cells without sgRNA. Average and standard deviation for three technical replicates are shown. **d**, Calu-3 CRISPRi cells expressing different sgRNAs targeting hit genes were infected with SARS-CoV-2 and viral RNA in the supernatant measured by RT-qPCR as a function of time post-infection. Average and standard deviation of three wells are shown. **e**, Viral RNA as measured by RT-qPCR at each time point. Average and standard deviation of three wells, same data as in (d).

Since *ACE2* was the top hit gene knockdown of which decreased Spike-RBD binding in our screen, we hypothesized that other hit genes might act by modulating ACE2 levels. Western Blots for ACE2 levels in Calu-3 cell lines expressing sgRNAs against validated target genes (hereafter referred to as knockdown lines) revealed marked changes in ACE2 protein levels. For hits associated with lower levels of Spike-RBD binding in the primary screen, we observed lower levels of ACE2 protein, and vice-versa for those hits associated with higher levels of Spike-RBD binding (Fig. 2a,b). To distinguish whether hit genes affected ACE2 protein levels via transcriptional or post-transcriptional mechanisms, we performed qPCR to measure *ACE2* transcript levels in these same knockdown lines. For all tested genes, we observed changes in *ACE2* transcript levels that were concordant with the changes in ACE2 protein levels (Fig. 2c), indicating that they acted on the transcriptional level. Some genes, such as *COMP* and *TRAPP*, showed relatively modest effects on ACE2 transcript levels, but quite large effects on ACE2 protein levels, suggesting that these hit genes additionally affect post-transcriptional regulation of ACE2 expression.

We next determined the effect of hit gene knockdown on susceptibility to SARS-CoV-2 infection. We infected cells expressing sgRNAs against hit genes with SARS-CoV-2 and measured virus replication 24, 48 and 72 hours post-infection using RT-qPCR (Fig. 2d,e). Already at 24 hours post-infection, viral genome copies diverged concordantly with changes in ACE2 levels and Spike-RBD binding: sgRNAs that lowered Spike-RBD binding reduced virus replication, while sgRNAs that increased Spike-RBD binding resulted in higher virus replication. Remarkably, *BRD2* knockdown completely abrogated viral replication in these cells to similar levels as ACE2 knockdown, even at 72 hours post-infection.

### BRD2 inhibitors prevent SARS-CoV-2 infection of human cells

Given the stringent inhibition of SARS-CoV-2 infection achieved by BRD2 knockdown, we decided to focus on this gene. BRD2 belongs to the bromodomain and extraterminal (BET) family of proteins, and is currently being evaluated as therapeutic target in cancer^22,23^, with several small molecule inhibitors in clinical trials^24^.

**Extended Data Figure 2:**
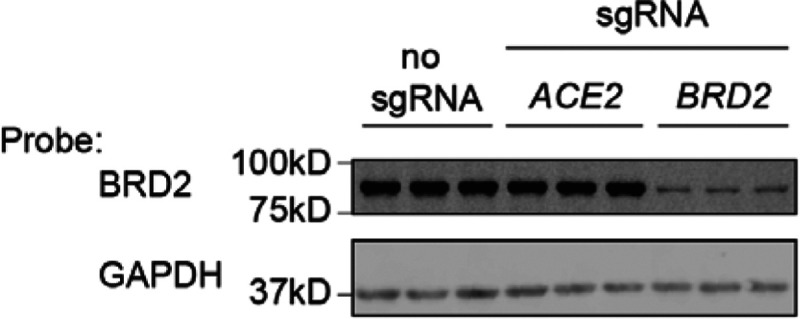
BRD2 is effectively knocked down by CRISPRi Western blot for BRD2 and the loading control GAPDH in CRISPRi Calu-3 cells expressing no sgRNA or sgRNAs targeting ACE2 or BRD2. Three lanes represent samples from three independent wells.

**Figure 3:**
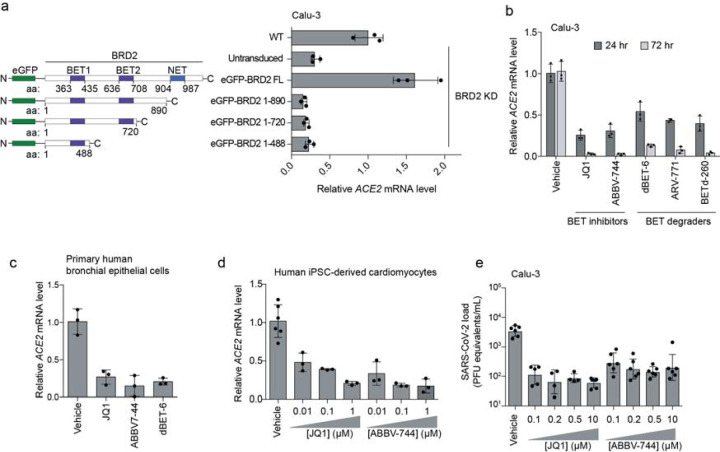
BRD2 inhibitors potently reduce ACE2 levels and SARS-CoV-2 infection **a**, Transgenic constructs expressed in Calu-3 cells (left). Transcript levels of ACE2 relative to ACTB in Calu3 cells transduced with eGFP-BRD2 truncations in a BRD2 knockdown background (right). ACE2 expression is relative to WT. Average and standard deviation of technical triplicates are shown for each transduced construct. **b**, Transcript levels of ACE2 relative to ACTB in Calu3 cells treated with BRD2 inhibitors (JQ1 at 10 μM, ABBV-744 at 10 μM and dBET-6 at 200 nM) were quantified at 24 (dark grey bars) and 72 hours (light grey bars) post-treatment. Average and standard deviation of technical triplicates are shown for each condition. **c**, Transcript levels of ACE2 relative to ACTB in primary human bronchial epithelial cells treated with BRD2 inhibitors (JQ1 at 10 μM, ABBV-744 at 1 μM and dBET-6 at 20 nM) were quantified at 72 hours post-treatment. Average and standard deviation of technical triplicates are shown for each condition. **d**, Transcript levels of ACE2 relative to 18S rRNA in human iPSC-derived cardiomyocytes treated with the indicated concentrations of BRD2 inhibitors were quantified at 72 hours post-treatment. Average and standard deviation of 3 or more biological replicates are shown for each condition. **e**, SARS-CoV-2 viral RNA in the supernatant measured by RT-qPCR 24 hours post-infection of Calu-3 cells infected 72 hours after treatment with the indicated concentrations of BRD2 inhibitors. Average and standard deviation of four or more biological replicates are shown for each condition.

We validated that CRISPRi knockdown of BRD2 robustly reduced BRD2 protein levels (Extended Data Fig. 2a). Over-expression of full-length BRD2 restored ACE2 transcript levels (Fig. 3a), validating that the reduction in ACE2 expression triggered by CRISPRi targeting of BRD2 was indeed due to BRD2 knockdown. Truncation mutants of BRD2 did not rescue ACE2 expression (Fig. 3a), indicating that full-length BRD2 is required for ACE2 expression.

**Extended Data Figure 3:**
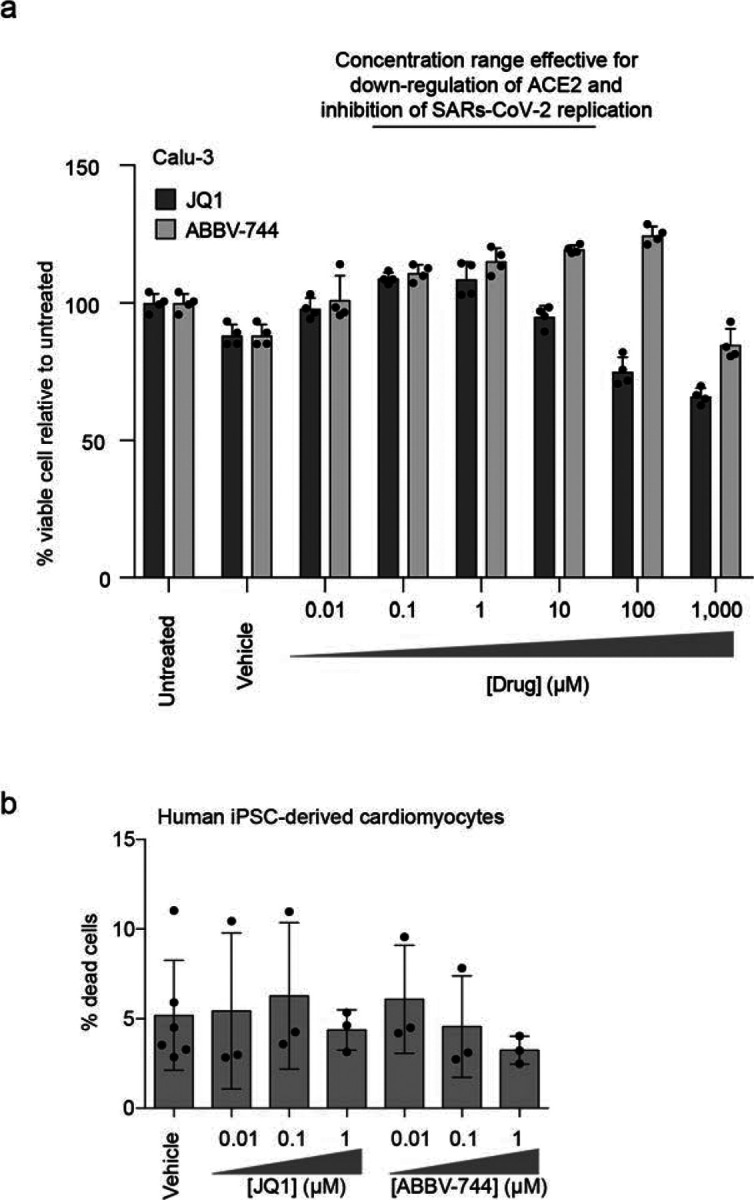
Non-toxic concentration range of BRD2 inhibitors **a**, Calu-3 cells were treated with vehicle or the indicated concentrations of JQ1 or ABBV-744 for 5 days. Cell viability was then assayed with CellTiter-Glo 2.0 to calculate viability. **b**, Human iPSC-derived cardiomyocytes were treated for 72 hours with vehicle or the indicated concentrations of JQ1 or ABBV-744, and the percentage of dead cells was quantified as the ratio of propidium iodide-positive cells (dead cells) over Hoechst-positive cells (all cells).

To test the potential of BRD2 as a therapeutic target for COVID-19, we treated cells with a panel of compounds targeting BRD2: two BET domain inhibitors, (JQ1^25^ and ABBV-744^26^, which is currently in clinical trials NCT03360006 and NCT04454658), and three Proteolysis Targeting Chimeric (PROTAC) compounds that lead to the degradation of BRD2 (dBET-6^27^, ARV-771^28^, and BETd-260^28^). After only 24 hours of treatment with these drugs, Spike-RBD binding was already decreased by at least two-fold. ACE2 mRNA levels measured by qPCR showed similar decreases (Fig. 3b). This effect was magnified after treatment for 72 hours, when almost no ACE2 mRNA was detectable for any of the BRD2-targeting compounds tested, phenocopying BRD2 knockdown (Fig. 3b). Similarly, we found that BET inhibitors led to substantial decreases in ACE2 mRNA levels in primary human bronchial epithelial cells (Fig. 3c) and human iPSC-derived cardiomyocytes (Fig. 3d), two non-transformed cell types that are susceptible to SARS-CoV-2 infection^29,30^. Importantly, BET inhibitors were non-toxic to Calu-3 cells and cardiomyocytes at effective concentrations (Extended Data Fig. 3).

Since pharmacological inhibition of BRD2 phenocopied BRD2 knockdown, we hypothesized that these same compounds might prevent infection of cells exposed to SARS-CoV2. To test this, we treated Calu-3 cells for 72 hours with the BET inhibitors JQ-1 and ABBV-744, as BET inhibitors are already in clinical trials, and measured SARS-CoV-2 replication at 48 hours post infection. Strikingly, we found that treated cells displayed 100-fold decreased viral replication versus untreated cells (Fig. 3e), a similar effect size to *BRD2* or *ACE2* knockdown (Fig. 2d,e).

### BRD2 regulates the transcription of ACE2 and other host genes induced by SARS-CoV-2 infection

**Figure 4:**
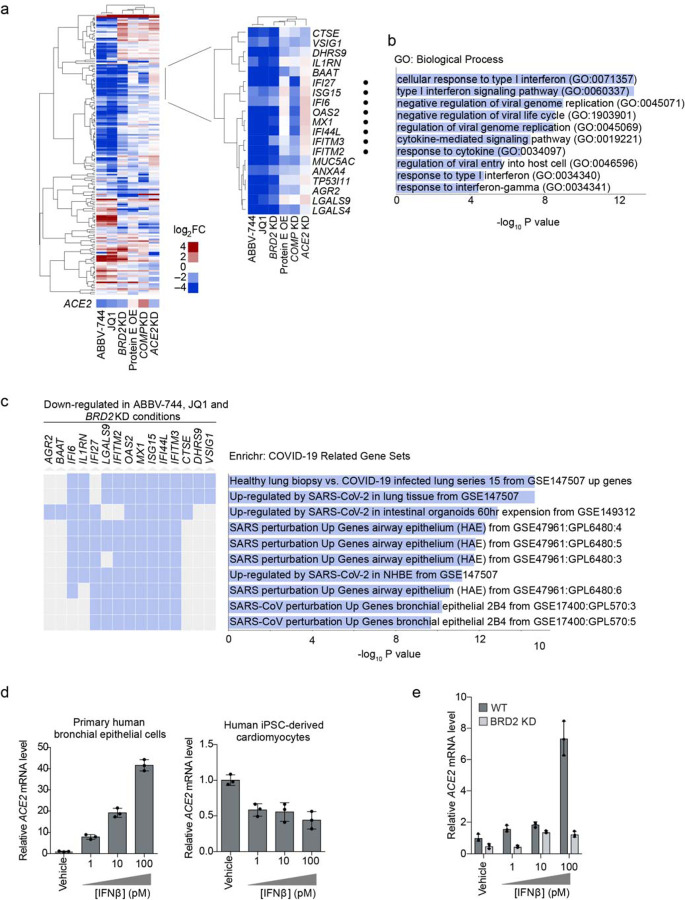
BRD2 controls genes induced by interferon and SARS-CoV-2 infection **a**, Differentially expressed genes from RNA sequencing of Calu-3 cells under different treatment conditions compared to control cells: 72-hour treatment with 10 μM JQ1 or 10 μM ABBV-744, *BRD2* knockdown (KD), SARS-CoV-2 protein E overexpression (OE), *COMP* KO, *ACE2* KD. Heatmap showing log_2_-fold change (log_2_FC) for each condition relative to untreated controls (columns) for genes that are among top 50 differentially expressed genes (ranked by P values) in at least one of the conditions (rows). ACE2 was not among these genes and is shown as a separate row. *Insert*, a cluster of genes that are down-regulated in both BRD2 inhibition by JQ1 and ABBV-744 and *BRD2* knockdown. Among these, genes associated with the GO term “Cellular Response to Type I interferon” are marked by black dots. **b**, Significantly enriched (FDR < 0.05) GO biological process terms for the genes shown in the inset in (a). **c**, Enrichment analysis for genes in the inset in (a) reveals COVID-19 related gene sets. Genes that appear in a gene set are marked in blue. **d**, Primary human bronchial epithelial cells (left) and human iPSC-derived cardiomyocytes (right) were treated with the indicated concentrations of interferon-beta (IFNβ), and transcript levels of ACE2 relative to ACTB (for Calu-3 and NHBE) or 18S rRNA (cardiomyocytes) were quantified at 72 hours post-treatment by qPCR. Average and standard deviation of 3 technical replicates are shown for each condition. **e**, WT (dark grey) or BRD2 knockdown (light grey) Calu-3 cells were treated with the indicated concentrations of interferon-beta (IFNβ), and transcript levels of ACE2 relative to ACTB were quantified at 72 hours post-treatment by qPCR. Average and standard deviation of 3 technical replicates are shown for each condition.

We next asked whether BRD2 controls transcription of additional genes beyond ACE2. We performed RNA sequencing of Calu-3 cells after treatment with the BET-domain inhibitors JQ-1 and ABBV-744 as well as *BRD2* CRISPRi knockdown (Extended Data Table 2). We also included CRISPRi knockdown of two other validated hit genes from our screen, *COMP* and *ACE2*. RNA-seq of BRD2 knockdown and BET domain inhibitor treated cells recapitulated downregulation of *ACE2* (Fig. 4a). Surprisingly, the same treatments also resulted in marked downregulation of genes involved in the type I interferon response, while *ACE2* knockdown slightly increased expression of those same genes (Fig. 4a,b). Furthermore, the genes downregulated by both BRD2 knockdown and inhibition were strongly enriched in genes induced by SARS-CoV-2 infection in patient and cultured cells (Fig. 4c).

These findings are compatible with two distinct mechanisms: BRD2 could be a direct transcriptional regulator of *ACE2* and SARS-CoV-2-induced interferon response genes, or BRD2 could control interferon response genes, which in turn regulate *ACE2* transcription. In support of the latter mechanism, recent transcriptional studies of COVID-19 patients suggested that *ACE2* expression is induced by interferons^1,2^. Other studies, however, suggest that interferon suppresses *ACE2* expression^3^.

We found that treatment with IFNβ, a type I interferon, strongly increased *ACE2* mRNA levels in primary human bronchial epithelial cells but reduced *ACE2* mRNA levels in human iPSC-derived cardiomyocytes (Fig. 4d), suggesting that the effect of interferons on ACE2 is context-dependent, and BRD2 inhibition is likely to affect *ACE2* transcription through mechanisms other than reduced interferon gene expression.

To determine the pathway relationship between BRD2 and IFNβ in controlling *ACE2* expression, we treated WT and *BRD2* KD Calu-3 cells with IFNβ. IFNβ induced ACE2 expression in Calu-3 cells but not in BRD2 KD cells (Fig. 4e). This finding is incompatible with a model in which interferon response genes mediate *ACE2* induction downstream of BRD2, and suggests that BRD2 might directly control *ACE2* transcription.

**Figure 5:**
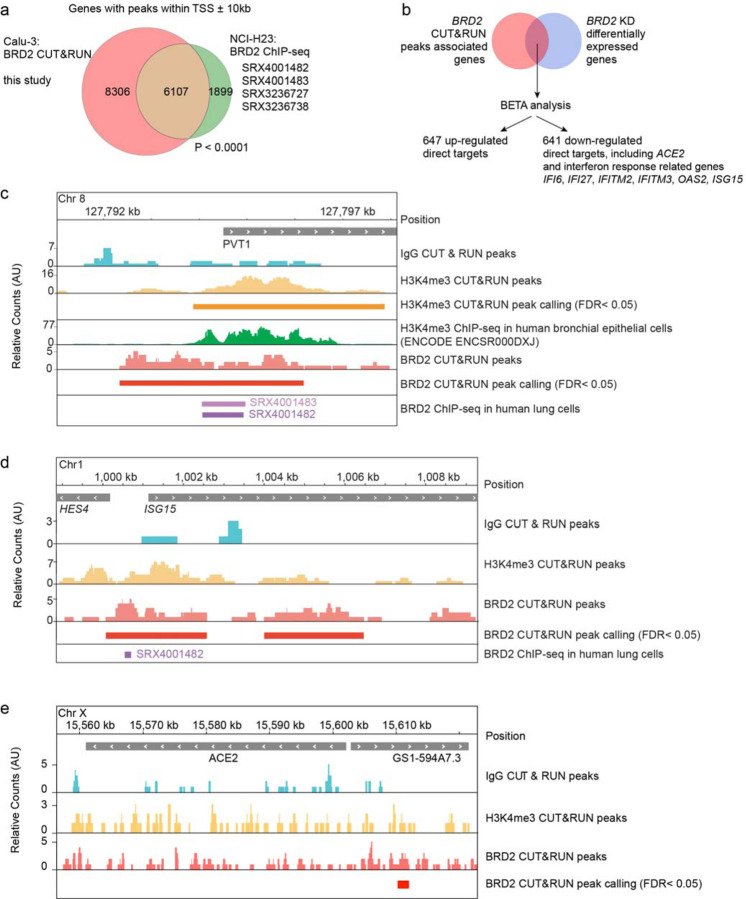
BRD2 directly regulates transcription of ACE2 and interferon-induced genes **a**, Genes associated with BRD2 CUT&RUN peaks within 10kb of a transcription start site determined in this study in Calu-3 cells overlap significantly with published BRD2 ChIP-seq peaks from the indicated datasets (P < 0.0001, Fisher’s exact test). **b**, Binding and Expression Target Analysis (BETA) was performed to identify direct BRD2 targets that were differentially expressed upon BRD2 knockdown. *ACE2* and several interferon response genes were identified as direct BRD2 targets. **c**, CUT&RUN recapitulates known BRD2 regulatory sites. Raw signal tracks for IgG, H3K4me3 and BRD2 are shown. Previously reported H3K4me3 CHIP-seq tracks as well as BRD2 CHIP-seq tracks from human lung cells are shown. BRD2 peaks were called over IgG using SEACR at FDR < 0.05. **d**, A BRD2 CUT&RUN peak was identified upstream of the interferon-induced gene *ISG15*. Raw signal tracks for IgG, H3K4me3 and BRD2 are shown. BRD2 peaks were called over IgG using SEACR at FDR < 0.05. BRD2 CHIP-seq tracks from human lung cells are also shown. **e**, A BRD2 CUT&RUN peak was identified within 10 kb upstream of *ACE2*. Raw signal tracks for IgG, H3K4me3 and BRD2 are shown. BRD2 peaks were called over IgG using SEACR at FDR < 0.05.

To test if BRD2 is indeed a direct transcriptional regulator of *ACE2*, we performed CUT&RUN^31^ to comprehensively map genomic loci bound by BRD2 in Calu-3 cells (Extended Data Table 3). Genes adjacent to BRD2-bound sites detected in our experiment showed a highly significant overlap with BRD2 sites previously mapped by ChIP-seq in NCI-H23^32^ cells, another lung epithelium-derived cancer cell line (Fig. 5a).

We performed Binding and Expression Target Analysis^33^ (BETA) to uncover direct BRD2 targets that were differentially expressed upon BRD2 knockdown, and identified *ACE2* and several interferon response genes as direct BRD2 targets (Fig. 5b). To validate our CUT&RUN results, we verified that a previously described^32^ BRD2 binding side upstream of *PVT1* was also detected in our experiment (Fig. 5c). We also mapped a BRD2 binding site upstream of the interferon response gene *ISG15* (Fig. 5d), and within 10 kb upstream of the *ACE2* transcription start site (Fig. 5e). These results support a model in which BRD2 directly controls *ACE2* transcription, as well as the transcription of interferon response genes, which can in turn additionally induce *ACE2* transcription in some cell types.

## Discussion

Here, we demonstrate that BRD2 is necessary for *ACE2* expression in cell lines representing lung epithelial cells and cardiomyocytes, cell types most vulnerable to SARS-CoV-2, and that BRD2 inhibition can block SARS-CoV-2 infection of the Calu-3 lung epithelial cell line. Our findings suggest that pharmacological BRD2 inhibitors may be of therapeutic benefit to prevent or reduce the impact of SARS-CoV-2 infection. One of the drugs we found to block SARS-CoV-2 infection in a cell-based model, ABBV-744, is currently in Phase I clinical trials (NCT03360006 and NCT04454658). Depending on its safety profile and side effects determined in those trials, ABBV-744 could be a candidate therapeutic for treatment after acute SARS-CoV-2 exposure.

We found that BRD2 is likely a direct transcriptional regulator of *ACE2* in COVID-19-relevant cell types. In Calu-3 cells, BRD2 is also a potent regulator of genes induced by SARS-CoV-2 infection, especially type I interferon response genes. In support of BRD2 as a direct regulator of both ACE2 and interferon-stimulated genes (ISGs), BRD2 has been shown to directly bind to- and assist in Histone H2A.Z removal from ISG promoters^34^. Taken together, this indicates that BRD2 could be a key regulator of the host response to SARS-CoV-2 infection.

The previously described^35^ interaction between the SARS-CoV-2 E protein and BRD2 might have evolved to manipulate gene expression during infection, including the expression of ACE2. In isolation, however, protein E overexpression in Calu-3 cells only partially recapitulated expression changes resulting from BRD2 knockdown or inhibition (Fig 4a). These data suggest that the effect of Protein E on BRD2 may be more nuanced, or that additional viral and/or host factors expressed during SARS-CoV-2 infection are required to modulate BRD2 function. Further studies are needed to define the function of the protein E-BRD2 interaction.

Several previous CRISPR screens aiming to uncover strategies to inhibit SARS-CoV-2 infection were carried out in cell lines in which an *ACE2* transgene was overexpressed^9,10^; these screens therefore failed to uncover BRD2 as a regulator of endogenous *ACE2* expression. BRD2 did show a phenotype, however, in a CRISPR screen carried out in Vero-E6 cells (which express ACE2 endogenously)^12^, although it was not further characterized in that study. Based on the observation that BRD2 physically interacts with the SARS-CoV-2 protein E, BET inhibitors JQ-1 and ABBV-744 were tested for their effect on SARS-CoV-2 replication in Vero-E6 cells^35^, but not found to be effective. Here, we found them to be potent restrictors of SARS-CoV-2 replication in Calu-3 cells. Together, these differences highlight the importance of investigating SARS-CoV-2 interactions with disease-relevant cell types.

There is a growing literature about the relationship between COVID-19 disease severity, ACE2 expression, and interferon regulation^1–6^. Since ACE2 is known to promote recovery after lung injury and that SARS-CoV-2 manipulates the host interferon response^36–38^, the mis-regulation of these two pathways may play a major role in enhancing the severity of COVID-19. Our data suggest that BRD2 is central to this regulatory network and, therefore, pharmacological targeting of BRD2 may be a promising therapeutic strategy for the treatment of COVID-19: BRD2 inhibition could both block viral entry, through ACE2 downregulation, and act as an “emergency-brake” for mis-regulated patient immune responses to COVID-19, via down-regulation of ISGs.

## Methods

### Cell Culture

Calu-3 cells were cultured in RPMI 1640 (Life Technologies 22400–105) with 10% FBS (VWR 89510–186), 1% Pen/Strep (Life Technologies 15140122), and 5 mM Glutamine (Life Technologies 25030081) at 37 °C and 5% CO_2_ Cells were split by treating with TrypLE (Life Technologies 12604013) for 15 minutes, quenching with media and spun down at 200×g for 5 minutes. At Institut Pasteur, where virus infections were carried out, Calu-3 cells were cultured in MEM (Gibco 11095–080) with 20% FBS (Gibco A3160801), 1% Pen/Strep (Gibco 15140–122), 1% NEAA (Sigma-Aldrich M7145) and 1 mM Sodium pyruvate (Sigma-Aldrich S8636). They were split in Trypsin-EDTA 0.05% (Gibco 11580626).

HEK293 cell culture and production of lentivirus was performed as previously described^39^.

A vial of STR authenticated Caco-2 cells was obtained from the UCSF Cell and Genome Engineering Core (CGEC). Caco-2 cells were cultured in EMEM (ATCC, 30–2003) with 20% FBS (VWR 89510–186), 1% Pen/Strep (Life Technologies 15140122), and 5 mM Glutamine (Life Technologies 25030081) at 37 °C and 5% CO2.

A vial of A549 cells was obtained from Davide Ruggero’s lab as a gift. A549 cells were cultured in DMEM (Thermo Fisher Scientific, 10313–039) with 10% FBS (VWR 89510–186), 1% Pen/Strep (Life Technologies 15140122), and 5 mM Glutamine (Life Technologies 25030081) at 37 °C and 5% CO2.

Human iPSC-derived cardiomyocytes were generated and cultured as previously described^29^, from AICS90 iPSCs (Allen Institute Cell Catalog). Drugs were added on day 69 of differentiation, and cardiomyocytes were harvested for analysis on day 72.

Normal human bronchial epithelia (Mattek NHBE-CRY) were cultured following the supplier’s instructions.

### Generation of the Calu-3 ACE2 knockout line

The polyclonal ACE2 knockout Calu-3 cell line was generated using the Gene KO kit V2 from Synthego, using three sgRNAs targeting ACE2 with the following protospacer sequences sRNA1: 5’-GACAUUCUCUUCAGUAAUAU-3’, sgRNA2: 5’-AAACUUGUCCAAAAAUGUCU-3’ and sgRNA3: 5’-UUACAGCAACAAGGCUGAGA-3’. Single guide RNAs (sgRNAs) were designed according to Synthego’s multiguide gene knockout kit^40^. Briefly, two or three sgRNAs are bioinformatically designed to work in a cooperative manner to generate small, knockout causing, fragment deletions in early exons. These fragment deletions are larger than standard indels generated from single guides. The genomic repair patterns from a multiguide approach are highly predictable on the basis of the guide spacing and design constraints to limit off-targets, resulting in a higher probability protein knockout phenotype.

The ribonucleoprotein (RNP) complex with a ratio of 4.5 to 1 between sgRNA and Cas9 was delivered following the protocol of the SE Cell Line 4D-NucleofectorTM X Kit (Lonza, V4XC-1012), using the nucleofection program DS-130 on the Lonza 4D X unit. 72 hours post transfection, genomic DNA was extracted to serve as the template for PCR amplification of the region that covers the sites targeted by the sgRNAs with the following two primers: ACE2-F: 5’-CTGGGACTCCAAAATCAGGGA-3’ and ACE2-R: 5’-CGCCCAACCCAAGTTCAAAG-3’. Sanger sequencing reactions using the sequencing primer ACE2-seq: 5’-CAAAATCAGGGATATGGAGGCAAACATC-3’ were then performed, and the knockout efficiency was determined to be 80% via ICE software from Synthego^41^ (https://ice.synthego.com/#/).

### Generation of the Calu-3 CRISPRi line

The parental Calu-3 line was obtained from the UCSF Cell and Genome Engineering Core. Calu-3 cells were cultured at 37 °C with 5% CO2 in EMEM media containing 10% FBS, 100 units/ml streptomycin, 100 μg/ml penicillin, and 2 mM glutamine. To generate the CRISPRi lines, ~3×10^6^ cells were seeded into media containing lentiviral particles packaging dCas9-BFP-KRAB under a UCOE-SFFV promoter^42^. Five days post infection, BFP-positive cells were sorted using a BD Fusion. To validate the CRISPRi line, Calu-3-CRISPRi cells were transduced with lentiviral particles expressing non-targeting sgRNA (protospacer 5’-GCTCCCAGTCGGCACCACAG-3’) or *CD81*-targeting sgRNA (protospacer 5’-GGCCTGGCAGGATGCGCGG-3’). CD81 expression was measured 7 days post-transduction by dislodging cells with TrypLE and live cells were stained with APC-conjugated anti-human CD81 antibody (Biolegend 349509). CD81 expression was assessed on a BD LSRII with >90% of Calu3-CRISPRi cells with CD81 knocked down compared to a non-targeting sgRNA control.

### Spike-RBD binding assay

Recombinant biotinylated SARS-CoV2 spike Spike-receptor-binding domain with a C-terminal human IgG Fc domain fusion (referred to as Spike-RBD) was prepared as previously described^43^. Calu-3 cells were grown in 96-well flat bottom plates until >50% confluent. Media was aspirated and cells were washed once with PBS. Cells were then treated with TrypLE to release them from the plate, RPMI 1640 media was added to dilute TrypLE, and cells were pelleted by centrifugation at 200×g for five minutes. From this point on, all steps were carried out on ice. Cells were incubated in 3% BSA (Sigma Aldrich A7030) in DPBS (Sigma-Aldrich D8537) for 15 minutes to block and washed twice in 3% BSA in DPBS by centrifugation at 200×g for five minutes in v-bottom plates, followed by resuspension. Spike-RBD was diluted in 3% BSA to appropriate concentrations and incubated with cells for 30 minutes on ice. Cells were then washed twice with 3% BSA in DPBS and incubated with Anti-Strep PE-Cy7 (Thermofisher SA1012) at 5 μg/mL. Cells were washed twice and subjected to flow cytometry on a FACS Celesta in HTS mode. Cells were gated to exclude doublets and the median PE-Cy7 signal was calculated for each sample. The gating strategy is shown in Supplemental Figure 2. EC_50_ values and their 95% confidence intervals were calculated by fitting the RBD binding data into a Sigmoidal, 4PL model in Prism 6.

### CRISPRi Screen

Calu-3 cells were infected with the H1 CRISPRi sgRNA library^20^ as described^39^ and selected using treatment with 1 μg/mL puromycin for 3 days. After selection, cells were stained with 10 nM Spike-RBD as described above or for TFRC as previously described^39^ and subjected to FACS, where cells were sorted into top 30% and bottom 30% based on high and low expression of TFRC or Spike-RBD. Sorted populations were spun down at 200×g for five minutes and genomic DNA was isolated as described^39^. sgRNA cassettes were amplified by PCR and sequencing and analysis was performed as described^39^ but with an FDR of 0.1.

### Validation of screening hits

Individual sgRNAs were selected based on phenotypes in the primary screens and cloned into a lentiviral expression vector as described^39^. Protospacer sequences of these sgRNAs are provided in Extended Data Table 4. Cells expressing sgRNAs were selected using treatment with 1 μg/mL puromycin for 3–7 days.

### Drug treatments

Drugs (ABBV-744 Selleckchem S8723, JQ1 - Sigma Aldrich SML1524, dBET6 - Selleckchem S8762) were dissolved in DMSO or water as per manufacturer’s instructions. Cells were treated with drugs for 72 hours with media changes performed every 24 hours with media containing fresh drug.

### Interferon Treatments

Interferon Beta (R&D systems 8499-IF) was dissolved per the manufacturer’s instructions. Cells were treated with IFNβ for 72 hours with media changes performed every 24 hours with media containing fresh IFNβ.

### qPCR

qPCR was performed and analyzed as described^39^. Primers: *ACE2* forward: GGTCTTCTGTCACCCGATTT; *ACE2* reverse: CATCCACCTCCACTTCTCTAAC; *ACTB* forward: ACCTTCTACAATGAGCTGCG; *ACTB* reverse: CCTGGATAGCAACGTACATGG

### Western Blotting

Cells from one confluent well of a six-well plate were lysed in RIPA buffer plus c0mplete EDTA-free protease inhibitor tablets (Roche 11873580001) and spun for 10 minutes at 21,000×g at 4°C. The pellet was removed and a BCA assay (Thermofisher 23225) was performed on the remaining supernatant. Lysate volumes with equivalent protein content were diluted with SDS-PAGE loading dye and subjected to gel electrophoresis on 4–12% BisTris SDS-PAGE gels (Life Technologies NP0322) . Gels were then transferred and blocked in 5% NFDM for 1 hour at RT. Antibodies in fresh 5% NFDM were added (Mouse monoclonal GAPDH 1:10,000; Goat polyclonal ACE2 [R&D Tech AF933] 1:200; Rabbit monoclonal BRD2 [abcam 197865] 1:5,000) and incubated at 4 °C for at least 16 hours. Membranes were washed 4x with TBS + 0.1% Tween-20 and incubated with secondary antibodies (1:10,000 Donkey Anti-Goat-800 [LICOR 926–32214], LICOR Donkey Anti-Mouse-680 [LICOR 926–68072], HRP Donkey anti-rabbit [CST 7074P2]). Membranes were visualized using a LiCOR or Femto HRP kit (Thermofisher 34094). Uncropped images of Western blots are provided as Supplemental Figure 1.

### Virus

The SARS-CoV-2 strain used (BetaCoV/France/IDF0372/2020 strain) was propagated once in Vero-E6 cells and is a kind gift from the National Reference Centre for Respiratory Viruses at Institut Pasteur, Paris, originally supplied through the European Virus Archive goes Global platform.

### Cytotoxicity measurements of Calu3 cells

30,000 Calu-3 cells per well were seeded into Greiner 96-well white bottom plates and incubated for 48 hours at 37°C, 5% CO_2_. Then, cells were treated with identical drug concentrations as in the infection assays for 5 days by refreshing the media with 100μL per well fresh drug-containing media every 24 hours. Cell viability was then assayed by adding 100μL per well of CellTiter-Glo 2.0 (Promega) and incubated for 10 minutes at room temperature. Luminescence was recorded with an Infinite 200 Pro plate reader (Tecan) using an integration time of 1s.

### Virus infection assays

30,000 Calu-3 cells per well were seeded into 96-well plates and incubated for 48 hours at 37°C, 5% CO_2_. At the time of infection, the media was replaced with virus inoculum (MOI 0.1 PFU/cell) and incubated for one hour at 37°C, 5% CO_2_. Following the one-hour adsorption period, the inoculum was removed, replaced with fresh media, and cells incubated at 37°C, 5% CO_2_. 24h, 48h and 72h post infection, the cell culture supernatant was harvested, and viral load assessed by RT-qPCR as described previously^35^. Briefly, the cell culture supernatant was collected, heat inactivated at 95°C for 5 minutes and used for RT-qPCR analysis. SARS-CoV-2 specific primers targeting the N gene region: 5′-TAATCAGACAAGGAACTGATTA-3′ (Forward) and 5′-CGAAGGTGTGACTTCCATG-3′ (Reverse) were used with the Luna Universal One-Step RT-qPCR Kit (New England Biolabs) in an Applied Biosystems QuantStudio 6 thermocycler or an Applied Biosystems StepOnePlus system, with the following cycling conditions: 55°C for 10 min, 95°C for 1 minute, and 40 cycles of 95°C for 10 seconds, followed by 60°C for 1 minute. The number of viral genomes is expressed as PFU equivalents/mL, and was calculated by performing a standard curve with RNA derived from a viral stock with a known viral titer.

### CUT&RUN

*CUT&RUN* was performed with 1 million Calu-3 cells. Cells were removed from the plate by treatment with Versene (Life Technologies 15040066) for 20 minutes and resuspended in fresh media. They were spun down and washed twice with DPBS before proceeding with the CUTANA *CUT&RUN* kit (Epicypher 14–0050). The experiment was performed with the included IgG and H3K4Me control antibodies and the BRD2 antibody (abcam 197865) as well as *E.coli* spike-in DNA according to the kit protocol.

### QuantSeq analysis

Raw sequencing reads from QuantSeq were trimmed using Trimmomatic^44^ (v0.39, PMID: 24695404) and mapped to the human reference transcriptome (GRCh38, GENCODE Release 36) using Salmon^45^ (v1.3.0) to obtain transcript abundance counts. Gene-level count estimates were obtained using tximport^46^ (v1.18.0) with default settings. Subsequently, differential gene-expression analyses were performed using the glmQLFTest method implemented in the edgeR package^47^(v3.28.1). Cluster^48^ (v3.0) was used for hierarchical clustering and Java TreeView^49^ (v1.1.6r4) for visualization.

### CUT&RUN analysis

CUT&RUN analysis was performed as previously described^50^. Briefly, paired-end reads were mapped to the human genome GRCh38 using Bowtie2 (v2.3.4.1) with options: --end-to-end --very-sensitive --no-unal --no-mixed --no-discordant --phred33 -I 10 -X 1000. Sparse Enrichment Analysis for CUT&RUN (SEACR^51^, https://seacr.fredhutch.org/) was used for peak calling. H3K4me3 and BRD2 peaks were normalized to IgG control. Published BRD2 ChIP-seq data in human lung cells was obtained from ChIP-Atlas (https://chip-atlas.org/). The Integrative Genomics Viewer (IGV, igv.org) was used for visualization.

## Extended Data

## Supplementary Material

Supplement 1 **Extended Data Table 1: Phenotypes from CRISPRi screens for Spike-RBD and anti-TFRC binding.** Results from CRISPRi screens for Spike-RBD and anti-TFRC binding were analyzed by the MAGeCK-iNC pipeline (see Methods for details) and are listed for all genes targeted by the H1 sgRNA library. Columns are: Targeted gene, targeted transcription start site, knockdown phenotype (epsilon), P value, and Gene score.

Supplement 2 **Extended Data Table 2: Results from Quant-Seq experiments.** The first six tabs show the results of differential gene expression analyses for *ACE2* knockdown, ABBV-744 treatment, *BRD2* knockdown, JQ1 treatment, SARS-CoV-2 protein E overexpression and *COMP* knockdown, respectively, using edgeR (see Methods for details).
Columns are: Gene symbol, log_2_-fold change, log_2_ counts per million, F value, P value and FDR by the Benjamini-Hochberg method.
The ‘TPM’ tab shows the raw Transcripts Per Million (TPM) values for all samples. Columns: treatment conditions with 2 replicates each. Rows: all genes in the human transcriptome reference. The last tab provides the numerical values underlying the heatmap in Figure 4a. Columns: treatment conditions Rows: genes that are among top 50 differentially expressed genes in any of the conditions.

Supplement 3 **Extended Data Table 3: Results from CUT&RUN experiments.** BRD2 direct targets that are up- or down-regulated in the *BRD2* knockdown condition identified by the BETA analyses are listed. Columns are: chromosome, gene start position, gene end position, refseq ID, rank product, strand information and gene symbol.

Supplement 4 **Extended Data Table 4: Protospacer sequences of individually tested sgRNAs.** Protospacer sequences of individual sgRNAs used in Figure 1g are listed.

1

## References

[1] Ziegler C. G. K. SARS-CoV-2 Receptor ACE2 Is an Interferon-Stimulated Gene in Human Airway Epithelial Cells and Is Detected in Specific Cell Subsets across Tissues. Cell 181, 1016–1035.e19 (2020). 32413319 10.1016/j.cell.2020.04.035 PMC7252096

[2] Chu H. Comparative Replication and Immune Activation Profiles of SARS-CoV-2 and SARS-CoV in Human Lungs: An Ex Vivo Study With Implications for the Pathogenesis of COVID-19. Clin. Infect. Dis. 71, 1400–1409 (2020). 32270184 10.1093/cid/ciaa410 PMC7184390

[3] Gutiérrez-Chamorro L. SARS-CoV-2 infection suppresses ACE2 function and antiviral immune response in the upper respiratory tract of infected patients. bioRxiv 2020.11.18.388850 (2020). doi:10.1101/2020.11.18.388850

[4] Blanco-Melo D. Imbalanced Host Response to SARS-CoV-2 Drives Development of COVID-19. Cell 181, 1036–1045.e9 (2020). 32416070 10.1016/j.cell.2020.04.026 PMC7227586

[5] Bastard P. Autoantibodies against type I IFNs in patients with life-threatening COVID-19. Science. 370, eabd4585 (2020). 32972996 10.1126/science.abd4585 PMC7857397

[6] Hadjadj J. Impaired type I interferon activity and inflammatory responses in severe COVID-19 patients. Science. 369, 718–724 (2020). 32661059 10.1126/science.abc6027 PMC7402632

[7] Zhang Q. Inborn errors of type I IFN immunity in patients with life-threatening COVID-19. Science. 370, eabd4570 (2020). 32972995 10.1126/science.abd4570 PMC7857407

[8] Samuel R. M. Androgen Signaling Regulates SARS-CoV-2 Receptor Levels and Is Associated with Severe COVID-19 Symptoms in Men. Cell Stem Cell 27, 876–889.e12 (2020). 33232663 10.1016/j.stem.2020.11.009 PMC7670929

[9] Daniloski Z. Identification of Required Host Factors for SARS-CoV-2 Infection in Human Cells. Cell (2020). doi:10.1016/j.cell.2020.10.030 PMC7584921 33147445

[10] Wang R. Genetic Screens Identify Host Factors for SARS-CoV-2 and Common Cold Coronaviruses. Cell 1–14 (2020). doi:10.1016/j.cell.2020.12.004 PMC7723770 33333024

[11] Schneider W. M. Genome-Scale Identification of SARS-CoV-2 and Pan-coronavirus Host Factor Networks. Cell 184, 120–132.e14 (2021). 33382968 10.1016/j.cell.2020.12.006 PMC7796900

[12] Wei J. Genome-wide CRISPR Screens Reveal Host Factors Critical for SARS-CoV-2 Infection. Cell (2020). doi:10.1016/j.cell.2020.10.028 PMC7574718 33147444

[13] Lui I. Trimeric SARS-CoV-2 Spike interacts with dimeric ACE2 with limited intra-Spike avidity. bioRxiv 2020.05.21.109157 (2020). doi:10.1101/2020.05.21.109157

[14] Lan J. Structure of the SARS-CoV-2 spike receptor-binding domain bound to the ACE2 receptor. Nature 581, 215–220 (2020). 32225176 10.1038/s41586-020-2180-5

[15] Chua R. L. COVID-19 severity correlates with airway epithelium–immune cell interactions identified by single-cell analysis. Nat. Biotechnol. 38, 970–979 (2020). 32591762 10.1038/s41587-020-0602-4

[16] Tseng C.-T. K. Apical Entry and Release of Severe Acute Respiratory Syndrome-Associated Coronavirus in Polarized Calu-3 Lung Epithelial Cells. J. Virol. 79, 9470–9479 (2005). 16014910 10.1128/JVI.79.15.9470-9479.2005 PMC1181546

[17] Kuchi S., Gu Q., Palmarini M., Wilson S. J. & Robertson D. L. Meta-analysis of virus-induced host gene expression reveals unique signatures of immune dysregulation induced by SARS-CoV-2. bioRxiv 2020.12.29.424739 (2020). doi:10.1101/2020.12.29.424739

[18] Gilbert L. a CRISPR-Mediated Modular RNA-Guided Regulation of Transcription in Eukaryotes. Cell 154, 442–51 (2013). 23849981 10.1016/j.cell.2013.06.044 PMC3770145

[19] Gilbert L. A. Genome-Scale CRISPR-Mediated Control of Gene Repression and Activation. Cell 159, 647–661 (2014). 25307932 10.1016/j.cell.2014.09.029 PMC4253859

[20] Horlbeck M. A. Compact and highly active next-generation libraries for CRISPR-mediated gene repression and activation. Elife 5, 1–20 (2016). 10.7554/eLife.19760 PMC5094855 27661255

[21] Deffieu M. S. CRISPR-based bioengineering of the Transferrin Receptor revealed a role for Rab7 in the biosynthetic secretory pathway. bioRxiv 2020.01.05.893206 (2020). doi:10.1101/2020.01.05.893206

[22] Shi J. & Vakoc C. R. The Mechanisms behind the Therapeutic Activity of BET Bromodomain Inhibition. Mol. Cell 54, 728–736 (2014). 24905006 10.1016/j.molcel.2014.05.016 PMC4236231

[23] Doroshow D. B., Eder J. P. & LoRusso P. M. BET inhibitors: a novel epigenetic approach. Ann. Oncol. 28, 1776–1787 (2017). 28838216 10.1093/annonc/mdx157

[24] Xu Y. & Vakoc C. R. Targeting Cancer Cells with BET Bromodomain Inhibitors. Cold Spring Harb. Perspect. Med. 7, a026674 (2017). 28213432 10.1101/cshperspect.a026674 PMC5495050

[25] Filippakopoulos P. Selective inhibition of BET bromodomains. Nature 468, 1067–1073 (2010). 20871596 10.1038/nature09504 PMC3010259

[26] Faivre E. J. Selective inhibition of the BD2 bromodomain of BET proteins in prostate cancer. Nature 578, 306–310 (2020). 31969702 10.1038/s41586-020-1930-8

[27] Winter G. E. BET Bromodomain Proteins Function as Master Transcription Elongation Factors Independent of CDK9 Recruitment. Mol. Cell 67, 5–18.e19 (2017). 28673542 10.1016/j.molcel.2017.06.004 PMC5663500

[28] Shi C. PROTAC induced-BET protein degradation exhibits potent anti-osteosarcoma activity by triggering apoptosis. Cell Death Dis. 10, 815 (2019). 31653826 10.1038/s41419-019-2022-2 PMC6814818

[29] Pérez-Bermejo J. A. SARS-CoV-2 infection of human iPSC-derived cardiac cells predicts novel cytopathic features in hearts of COVID-19 patients. bioRxiv 2020.08.25.265561 (2020). doi:10.1101/2020.08.25.265561 PMC8128284 33723017

[30] Mulay A. SARS-CoV-2 infection of primary human lung epithelium for COVID-19 modeling and drug discovery. bioRxiv 2020.06.29.174623 (2020). doi:10.1101/2020.06.29.174623 PMC8043574 33905739

[31] Skene P. J. & Henikoff S. An efficient targeted nuclease strategy for high-resolution mapping of DNA binding sites. Elife 6, 1–35 (2017). 10.7554/eLife.21856 PMC5310842 28079019

[32] Handoko L. JQ1 affects BRD2-dependent and independent transcription regulation without disrupting H4-hyperacetylated chromatin states. Epigenetics 13, 410–431 (2018). 30080437 10.1080/15592294.2018.1469891 PMC6140815

[33] Wang S. Target analysis by integration of transcriptome and ChIP-seq data with BETA. Nat. Protoc. 8, 2502–2515 (2013). 24263090 10.1038/nprot.2013.150 PMC4135175

[34] Au-Yeung N. & Horvath C. M. Histone H2A.Z Suppression of Interferon-Stimulated Transcription and Antiviral Immunity Is Modulated by GCN5 and BRD2. iScience 6, 68–82 (2018). 30240626 10.1016/j.isci.2018.07.013 PMC6137307

[35] Gordon D. E. A SARS-CoV-2 protein interaction map reveals targets for drug repurposing. Nature 583, 459–468 (2020). 32353859 10.1038/s41586-020-2286-9 PMC7431030

[36] Ribero M. S., Jouvenet N., Dreux M. & Nisole S. Interplay between SARS-CoV-2 and the type I interferon response. PLoS Pathog. 16, 1–22 (2020). 10.1371/journal.ppat.1008737 PMC7390284 32726355

[37] Lei X. Activation and evasion of type I interferon responses by SARS-CoV-2. Nat. Commun. 11, 3810 (2020). 32733001 10.1038/s41467-020-17665-9 PMC7392898

[38] Xia H. Evasion of Type I Interferon by SARS-CoV-2. Cell Rep. 33, 108234 (2020). 32979938 10.1016/j.celrep.2020.108234 PMC7501843

[39] Tian R. CRISPR Interference-Based Platform for Multimodal Genetic Screens in Human iPSC-Derived Neurons. Neuron 104, 239–255.e12 (2019). 31422865 10.1016/j.neuron.2019.07.014 PMC6813890

[40] Stoner R., Maures T. & Conant D. Methods and Systems for guide RNA Design and Use. (2019). US Patent 16418893

[41] Hsiau T. Inference of CRISPR Edits from Sanger Trace Data. bioRxiv 251082 (2019). doi:10.1101/251082 35119294

[42] Adamson B. A Multiplexed Single-Cell CRISPR Screening Platform Enables Systematic Dissection of the Unfolded Protein Response. Cell 167, 1867–1882.e21 (2016). 27984733 10.1016/j.cell.2016.11.048 PMC5315571

[43] Glasgow A. Engineered ACE2 receptor traps potently neutralize SARS-CoV-2. Proc. Natl. Acad. Sci. 117, 28046–28055 (2020). 33093202 10.1073/pnas.2016093117 PMC7668070

[44] Bolger A. M., Lohse M. & Usadel B. Trimmomatic: A flexible trimmer for Illumina sequence data. Bioinformatics 30, 2114–2120 (2014). 24695404 10.1093/bioinformatics/btu170 PMC4103590

[45] Patro R., Duggal G., Love M. I., Irizarry R. A. & Kingsford C. Salmon provides fast and bias-aware quantification of transcript expression. Nat. Methods 14, 417–419 (2017). 28263959 10.1038/nmeth.4197 PMC5600148

[46] Soneson C., Love M. I. & Robinson M. D. Differential analyses for RNA-seq: transcript-level estimates improve gene-level inferences. F1000Research 4, 1521 (2016). 10.12688/f1000research.7563.1 PMC4712774 26925227

[47] Robinson M. D., McCarthy D. J. & Smyth G. K. edgeR: a Bioconductor package for differential expression analysis of digital gene expression data. Bioinformatics 26, 139–140 (2010). 19910308 10.1093/bioinformatics/btp616 PMC2796818

[48] Eisen M. B., Spellman P. T., Brown P. O. & Botstein D. Cluster analysis and display of genome-wide expression patterns. Proc. Natl. Acad. Sci. 95, 14863–14868 (1998). 9843981 10.1073/pnas.95.25.14863 PMC24541

[49] Saldanha A. J. Java Treeview--extensible visualization of microarray data. Bioinformatics 20, 3246–3248 (2004). 15180930 10.1093/bioinformatics/bth349

[50] Skene P. J., Henikoff J. G. & Henikoff S. Targeted in situ genome-wide profiling with high efficiency for low cell numbers. Nat. Protoc. 13, 1006–1019 (2018). 29651053 10.1038/nprot.2018.015

[51] Meers M. P., Tenenbaum D. & Henikoff S. Peak calling by Sparse Enrichment Analysis for CUT&RUN chromatin profiling. Epigenetics Chromatin 12, 42 (2019). 31300027 10.1186/s13072-019-0287-4 PMC6624997

