# Supplementary material for: BRD2 inhibition blocks SARS-CoV-2 infection in vitro by reducing transcription of the host cell receptor ACE2
Source: bioRxiv. 2021 Jan 19:2021.01.19.427194. Preprint. [Version 1] doi: 10.1101/2021.01.19.427194 (PMC7836110; doi:10.1101/2021.01.19.427194)
Supplement: Supplementary file 1 [file NIHPP2021.01.19.427194-supplement-1.pdf]

bioRxiv preprint doi: https://doi.org/10.1101/2021.01.19.427194; this version posted January 19, 2021. The copyright holder for this preprint (which was not certified by peer review) is the author/funder, who has granted bioRxiv a license to display the preprint in perpetuity. It is made available under aCC-BY 4.0 International license.

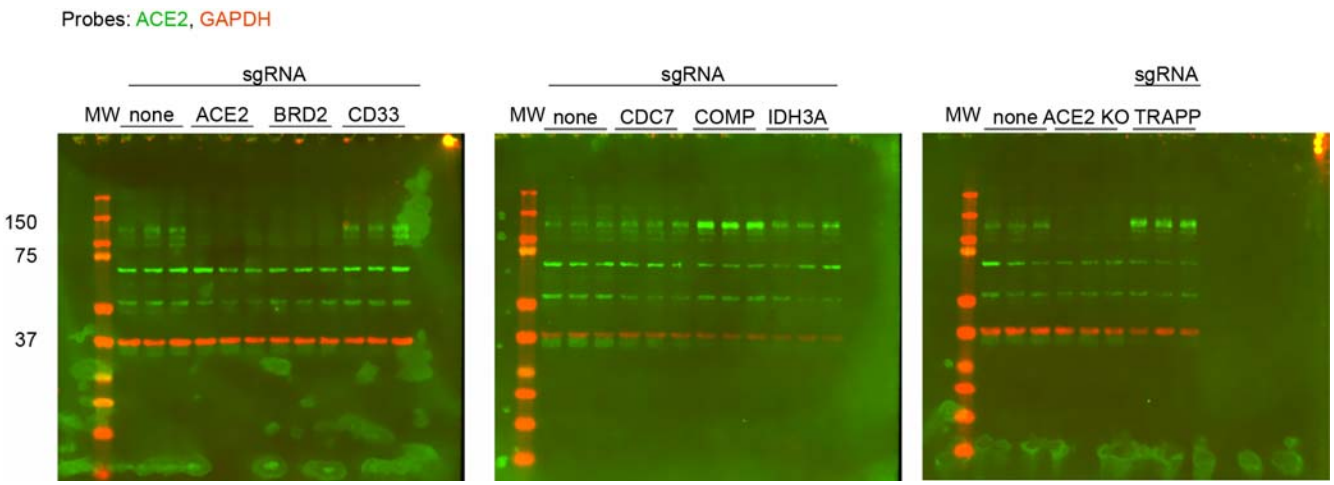

Supplemental Figure 1: Full-size Western blots, associated with Fig. 2a and Extended Data Fig. 2

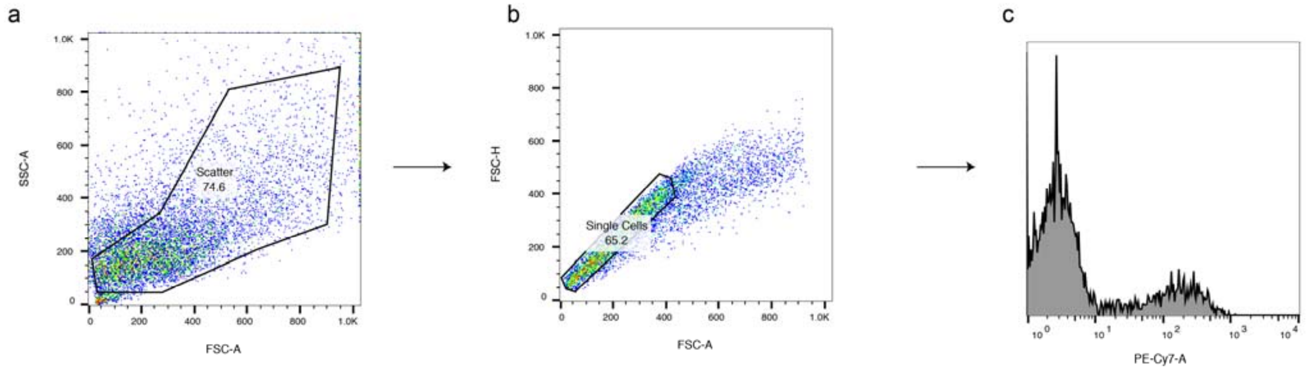

Supplemental Figure 2: Flow cytometry gating strategy, associated with Fig. 1
